# NAFLD Aggravates Septic Shock Due to Inadequate Adrenal Response and 11β-HSDs Dysregulation in Rats

**DOI:** 10.3390/pharmaceutics12050403

**Published:** 2020-04-28

**Authors:** Hui-Chun Huang, Ming-Hung Tsai, Fa-Yauh Lee, Te-Yueh Lin, Ching-Chih Chang, Chiao-Lin Chuang, Shao-Jung Hsu, Ming-Chih Hou, Yi-Hsiang Huang

**Affiliations:** 1 Faculty of Medicine, School of Medicine, National Yang-Ming University, Taipei 100, Taiwan; hchuang2@vghtpe.gov.tw (H.-C.H.); fylee@vghtpe.gov.tw (F.-Y.L.); ccchang7@vghtpe.gov.tw (C.-C.C.); clchuang@vghtpe.gov.tw (C.-L.C.); mchou@vghtpe.gov.tw (M.-C.H.); yhhuang@vghtpe.gov.tw (Y.-H.H.); 2 Division of Gastroenterology and Hepatology, Department of Medicine, Taipei Veterans General Hospital, Taipei 11217, Taiwan; derickskylark@gmail.com; 3 Division of General Medicine, Department of Medicine, Taipei Veterans General Hospital, Taipei 11217, Taiwan; 4 Division of Gastroenterology, Chang Gung Memorial Hospital, Chang Gung University, Taoyuan 320-338, Taiwan; mhtsai@cgmh.org.tw

**Keywords:** non-alcoholic fatty liver disease, adrenal function, 11β-HSD1, shock

## Abstract

Background: Non-alcoholic fatty liver disease (NAFLD) is linked with metabolic syndrome. Previous studies showed that obesity may disrupt adrenal function and adversely affect its counter-regulations against shock. This study hence evaluated adrenal function abnormalities in NAFLD with shock. Methods: Sprague-Dawley rats were fed with regular chow-diet (control) or high fat diet (HFD, 60% energy derived from fat). Blood tests were performed at the end of the 4th, 6th and 8th week, respectively. Experiments were performed at the end of the 8th week. Results: HFD rats developed NAFLD. HFD rats had 27% and 51% increase in plasma corticosterone at the 6th and 8th week in usual status. However, HFD rats had 5 times more reduction of mean arterial pressure in response to lipopolysaccharide-induced sepsis as compared to control rats. The corticosterone increment ratio was also lower in HFD rats, even after ACTH administration. 11β-HSD system tended to generate more corticosterone in HFD rats under hemodynamic stable status without shock and the trend was lost in HFD rats with septic shock. Conclusion: Rats with NAFLD had profound septic shock due to inadequate corticosterone response. This is, at least partly, due to 11β-HSDs dysregulation in sepsis.

## 1. Introduction

NAFLD (non-alcoholic fatty liver disease) and NASH (non-alcoholic steatohepatitis) are major global public health problems linked with diabetes, obesity, insulin resistance and metabolic syndrome. It has been reported that over 90% of obese patients develop NAFLD [1]. NAFLD is liver steatosis without excessive alcohol use and NASH is a form of NAFLD with liver inflammation and damage.

It is noteworthy that cortisol-adrenocorticotropic hormone (ACTH) feedback may be defective in obesity and male obesity patients suffer from stress-stimulated hypothalamus-pituitary-adrenal (HPA) axis dysfunction [2]. Furthermore, subjects with NAFLD had a significantly blunted suppression of cortisol secretion by a low dose of dexamethasone, suggesting the possibility of a subtle, chronic activation of the hypothalamic-pituitary-adrenal (HPA) axis [3]. Interestingly, overweight type 2 diabetes mellitus patients with NAFLD have a subtle, chronic over-activity of the HPA axis and cortisol overproduction that might be implicated in the development of hepatic steatosis [4]. The same group also published that overweight NAFLD patients have a subtle, chronic over-activity of the HPA axis leading to subclinical cortisol overproduction that might be implicated in the development of NAFLD [5].

The body usually combats critical illness via HPA axis activation, which is highlighted by increased serum corticotropin and cortisol levels [6]. This is crucial for the adaptation to stress, because cortisol inhibits inflammatory mediators [7] and potentiates vascular responses to vasoconstrictors such as catecholamine, angiotensin II and vasopressin [8]. However, critical illness–related corticosteroid insufficiency (CIRCI) do exist with a subnormal adrenal response to adrenocorticotropin in severe illness, in which the cortisol levels, even though high at their absolute value, are insufficient to deal with the stress. Consistently, a hydrocortisone supplement in septic patients restored the vascular hypo-reactivity and reduced the incidence and severity of organ failure. These hemodynamic benefits may result from inhibition of cytokines and vasodilators such as nitric oxide (NO) [9], in which the latter also mediates vascular hypo-reactivity in liver cirrhosis and portal hypertension. Interestingly, a recent survey indicated that portal hypertension was present in 25% of patients upon the diagnosis of NAFLD and was associated with the extent of steatosis [10]. Our group and others have also demonstrated that adrenal insufficiency is common in critically ill cirrhotic patients and carries prognostic significance [11,12]. However, whether CIRCI exists in subjects with fatty liver disease has not been investigated.

Isozymes of 11β-hydroxysteroid dehydrogenase (11β-HSD1, 11β-HSD2) play pivotal roles in adrenal function by interconverting cortisol and cortisone (in rodents, corticosterone and 11-dehydrocorticosterone, respectively), in which cortisol and corticosterone are active corticosteroids. 11β-HSDs modulate the amount of active corticosteroids in a tissue-specific manner. 11β-HSD1 catalyzes both dehydrogenation (cortisol and corticosterone to cortisone and 11-dehydrocorticosterone, respectively) and reverse oxo-reduction (cortisone and 11-dehydrocorticosterone to cortisol and corticosterone, respectively). Reductase activity can be maintained or induced via NADPH catalyzed by glucose-6-phosphate dehydrogenase (G6PD) [13]. On the other hand, 11β-HSD2 acts almost exclusively as a dehydrogenase then reduces concentrations of active glucocorticoids. It has been recognized that there was an enhanced adipose tissue 11β-HSD1 activity in men with fatty liver [14]. However, hepatic 11β-HSD1 gene expression does not seem to be involved in liver fat accumulation or the progression of fatty liver to NASH [15]. The tissue-specific distributions of 11β-HSDs and G6PD in obesity and fatty liver diseases and their relationship with CIRCI, nevertheless, is not clear.

Comparing to clinical studies, animal experiments are feasible ways to survey the mechanism. This study, therefore, aimed to use a high fat diet (HFD)-fed rat model of NAFLD with or without lipopolysaccharide (LPS)-induced septic shock to address the aforementioned aspects.

## 2. Materials and Methods

### 2.1. Animal Model

Six-week-old male Sprague-Dawley rats without significant difference in body weight (BW) were fed with either regular chow-diet (control group, laboratory autoclavable rodent diet 5010, LabDiet, St. Louis, MO, USA) or high fat with calories adjusted diet (HFD group, 60% high fat diet, TD. 06414, Harlan, Indianapolis, IN, USA) ad libitum. The body weight was recorded weekly.

The animal works were performed at Taipei Veterans General Hospital and had been approved by Taipei Veterans General Hospital Animal Committee (IACUC 2013-090, IACUC 2014-060, IACUC 2016-098, Approval Date: 8 July 2013, 6 June 2014, 21 June 2016, respectively). All animals received humane care according to the criteria outlined in the “Guide for the Care and Use of Laboratory Animals” prepared by the National Academy of Sciences and published by the National Institutes of Health (NIH publication 86-23, revised 1985).

### 2.2. Study Design

#### 2.2.1. Changes of Hemodynamics, Biochemistry Parameters and HPA Axis in Control and HFD Rats without Sepsis

Rats were fed with regular chow-diet as control group or HFD. Body weight was recorded weekly and blood samplings were performed at the end of the 4th, 6th and 8th week, respectively. At the end of the 8th week, rats were subjected to the following experiments: (A) body weight (BW), systemic and portal hemodynamic parameters measurement; (B) Plasma glucose, triglyceride, cholesterol, alanine aminotransferase (ALT), aspartate aminotransferase (AST), insulin, corticotropin-releasing hormone (CRH), ACTH, aldosterone and corticosterone concentrations measurement; (C) Determination of protein expressions of G6PD, 11β-HSD1 and 11β-HSD2 in visceral adipose tissue and adrenal gland.

#### 2.2.2. Hemodynamic Response, Corticosterone Response and 11β-HSDs System Protein Expressions in Control and HFD Rats with LPS-Induced Sepsis

At the end of the 8th week, after BW, systemic and portal hemodynamic parameters measurements and blood withdrawn for corticosterone levels determination. Control and HFD group were subdivided to receive isotonic saline (vehicle) or LPS (40 mg/kg, i.p.), respectively. The hemodynamic parameters were monitored continuously for 120 min. After that, visceral adipose tissue and adrenal gland were dissected for protein expression analyses.

#### 2.2.3. Hemodynamic and Corticosterone Response to ACTH Stimulation in Control and HFD Rats with LPS-Induced Sepsis

One hundred and twenty minutes after LPS injection, ACTH (100 µg/0.2 mL normal saline, i.v., A6303, Sigma-Aldrich, St. Louis, MO, USA) was administrated. Blood before and 30 min after ACTH stimulation was withdrawn for calculation of corticosterone increment ratio. The hemodynamic parameters were recorded during the 30 min. Corticosterone response shown as corticosterone increment ratio was defined as the difference of corticosterone concentrations before and after ACTH stimulation, divided by the corticosterone level before ACTH stimulation [16].

### 2.3. Hemodynamic Measurements

The rats were anesthetized with ketamine hydrochloride (Ketalar, i.m., 100 mg/Kg body weight). The right femoral artery was cannulated with a PE-50 catheter connecting to a Model P23XL transducer (Argon Medical Devices Inc., Frisco, TX, USA). Continuous recordings of mean arterial pressure (MAP) and heart rate (HR) were performed on a MP45 two channel data acquisition system (BIOPAC Systems Inc., Goleta, CA, USA). The data were recorded and analyzed by BIOPAC BSL 4 (BIOPAC). The abdomen was then opened with a mid-line incision and the superior mesenteric vein was cannulated with the soft needle of 20G intravenous indwelling cannula connected to a Model P23XL transducer. Continuous recording of portal pressure (PP) was performed on a MP45 two-channel data acquisition system. The data was recorded and analyzed by BIOPAC BSL 4.

The superior mesenteric artery (SMA) was identified at its aortic origin and a 5-mm segment was gently dissected free from surrounding tissue. An ultrasonic flow transducer was placed around the SMA and the flow was detected through a small animal blood flow meter (T206, Transonic Systems Inc., Ithaca, NY, USA).

Cardiac output (CO) was measured by thermodilution, as previously described [17]. Briefly, a thermistor was placed in the aortic arch just distal to the aortic valve and the thermal indicator (100 μL of normal saline) was injected into the right atrium through a PE-50 catheter. The aortic thermistor was connected to a Columbus Instruments Cardiotherm 500-AC-R (Columbus Instruments International Co., Columbus, OH, USA). Five thermo-dilution curves were obtained for each cardiac output measurement. The final value was obtained from the arithmetic mean of the data. Cardiac index (CI, mL/min/100 g BW) was calculated as CO per 100 g BW. Systemic vascular resistance (SVR, mmHg/mL/min/100 g BW) was calculated via dividing MAP by CI. SMA resistance (mmHg/mL/min/100 g BW) was calculated by (MAP-PP)/SMA flow per 100 g BW.

### 2.4. Western Blotting

Liver, visceral adipose tissue, kidney and adrenal gland were immediately frozen in liquid nitrogen and stored at −80 °C until required. The protein extracts were made by pulverization in grinder with liquid nitrogen, mixed with a ratio of 1 mL of lysis buffer (phosphate-buffered solution containing 1% Nonidet P-40, 0.5% sodium deoxycholate, 0.1% sodium dodecyl sulfate (SDS)) and 0.05% protease inhibitor cocktail solution (Roche Diagnostics GmbH, Penzberg, Germany) for each 100 mg powdered tissue sample. Protein concentration was determined for each sample by the Bradford method. An aliquot of 20–40 µg protein from each sample that dissolved in sample buffer (63 mmol/l of Tris-HCL, pH 6.8, containing 2% SDS, 10% glycerol, 5% 2-mercaptoethanol and 0.005% bomphenol blue) was separated on denaturing SDS-10% polyacrylamide gels by electrophoresis (Mini-PROTEAN^®^ 3 Cell, Bio-Rad Laboratories, Hercules, CA, USA). Prestained proteins markers (SDS-PAGE Standards, Bio-Rad) were used for molecular weight determinations. Proteins were then transferred to a polyvinylidenedifluoride membrane (Immum-BlotTM PVDF Membrane, Bio-Rad) by a semi-dry electroblotting system (Trans-Blot^®^ SD Semi-dry Electrophoretic Transfer Cell, Bio-Rad) for 1.5 h at 4 °C. To block non-specific binding, membranes were blocked for 30 min with 5% non-fat dry milk in TBS-T, pH 7.4 (25 mmol/L Tris base-137 mmol/L NaCl-2.7 mmol/L KCL-1% Tween 20). Blots were incubated with the primary antibody [11β-HSD1 from R&D Systems, Minneapolis, MN, USA (AF3397); 11β-HSD2 from Millipore, Merk KGaA, Darmstadt, Germany (AB1296); G6PD from GeneTex Inc., Irvine, CA, USA (GTX89073)], diluted with 5% non-fat dry milk in TBS-T then washed. After that, the blots were incubated with the secondary antibody diluted with 5% non-fat dry milk in TBS-T [peroxidase-conjugated donkey anti-goat IgG, 1:6000 dilution (HAF109, R&D) for 11β-HSD1 and rabbit anti-sheep IgG antibody, 1:6000 (AP147P, Millipore) for 11β-HSD2] and washed. Subsequent detection of the target proteins (approximately 36 kDa for 11β-HSD1, 43.7 kDa for 11β-HSD2 and 59 & 62 kDa for G6PD) were performed by enhanced chemi-luminescence (Millipore). With a computer-assisted video densitometer and digitalized software (BioSpectrum^®^ 600 Imaging System, UVP Upland, CA, USA), the blots were scanned, photographed then the signal intensity (integral volume) of the appropriate band was analyzed.

### 2.5. Plasma Endocrine and Biochemical Indices

Hemospasia from the heart was done at the 4th, 6th and 8th week of feeding at 2 to 4 PM, when the plasma levels were similar to the 24-h average level [18]. After blood sampling, the rats were fasting for 16–18 h, then hemospasia was executed again to collect the fasting blood samples. The samples were centrifuged at 3000 g for 10 min and the supernatant as plasma was taken. Plasma triglyceride, cholesterol, alanine aminotransferase (ALT) and aspartate aminotransferase (AST) concentrations were measured by an analyzer (Cobas C501, Roche Ltd., Basel, Switzerland); plasma insulin, aldosterone and corticosterone concentrations were analyzed by ELISA (10-1250-01, Mercodia AB, Uppsala, Sweden; EC3001-1, Assaypro LLC, St Charles, MO, USA; 10004377, Cayman Chemical, Ann Arbor, MI, USA). Plasma glucose concentrations were determined by a glucose meter (AlphaTRAK, Abbott, Chicago, IL, USA). The blood glucose, triglyceride, cholesterol and insulin levels were analyzed from the fasting blood samples and ALT, AST, CRH, ACTH, aldosterone and corticosterone levels were analyzed from the non-fasting blood samples at 2 to 4 PM. HOMA-IR (Homeostatic Model Assessment for Insulin Resistance), the index of insulin resistance, was calculated as insulin (µU/mL) × glucose (mg/L)/22.5.

### 2.6. Liver Histology

Liver paraffin sections (5 μm) were stained with Hematoxylin and Eosin for the evaluation of tissue architecture and damage. Oil red O staining was applied to survey the extent of liver steatosis.

### 2.7. Statistical Analysis

All results are expressed as mean ± S.D. Statistical analyses were performed using an unpaired Student’s t-test or ANOVA with the Least Significant Difference test as appropriate. Results were considered statistically significant at a two-tailed P-value of less than 0.05. The SPSS 21 statistical package for Windows (SPSS Inc., Chicago, IL, USA) was used.

## 3. Results

### 3.1. Changes of Hemodynamics, Biochemistry Parameters and HPA Axis in Control and HFD Rats without Sepsis

#### 3.1.1. NAFLD and Portal Hypertension Developed in HFD-Fed Rats

**Figure 1:**
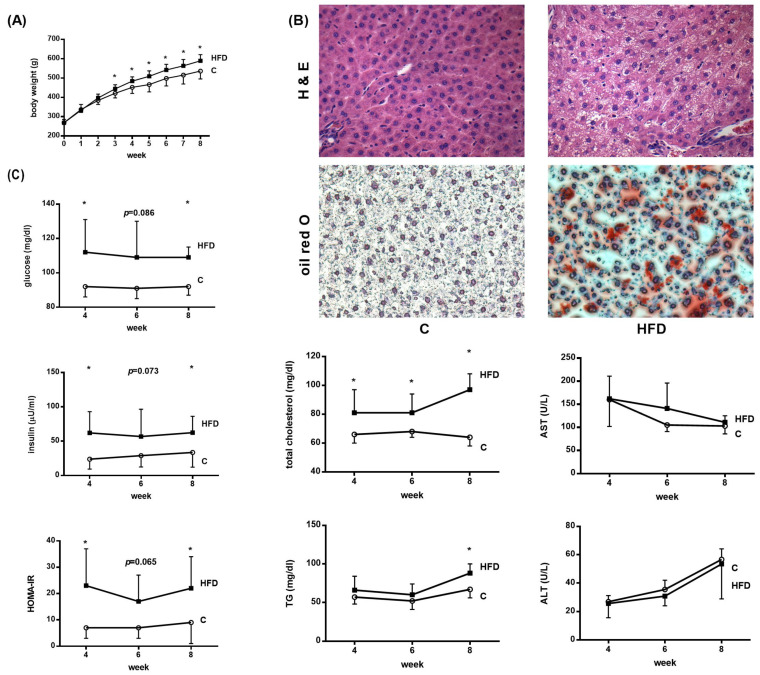
Effects of high fat diet (HFD) on liver biochemistry. Rats received regular chow diet (control, C) or HFD for 8 weeks. (**A**) Body weight increased significantly in HFD rats. (**B**) Liver histology showed marked ballooning change of hepatocytes and increased extent of oil red O staining, which is compatible with liver steatosis. (magnification: 20×) (**C**) The plasma glucose, insulin, HOMA-IR, cholesterol and triglyceride (TG) but not alanine aminotransferase (ALT), aspartate aminotransferase (AST) levels increased significantly in the HFD group. (* *p* < 0.05).

At the 8th week, BW increased significantly in HFD-fed rats (Figure 1A; control vs. HFD (g): 532 ± 21 v.s. 594 ± 10, *p* < 0.001, *n* = 8, 8). Liver histology showed ballooning change of hepatocytes and increased extent of oil red O staining, compatible with liver steatosis (Figure 1B). Figure 1C depicts the results of blood biochemistry. The levels of glucose, insulin and HOMA-IR, the index of insulin resistance, increased significantly in the HFD group (glucose (mg/dL): 92.2 ± 5.3 v.s. 108.9 ± 5.9, *p* < 0.001; insulin (μU/mL): 33.6 ± 21.4 v.s. 62.4 ± 23.8, *p* = 0.028; HOMA-IR (insulin × glucose): 9.1 ± 8.3 v.s. 21.7 ± 11.7, *p* = 0.030). The cholesterol and triglyceride levels also significantly increased in HFD group (cholesterol (mg/dL): 63.8 ± 6.0 v.s. 96.8 ± 11.3, *p* < 0.001; triglyceride (mg/dL): 66.6 ± 11.4 v.s. 88.4 ± 12.2, *p* = 0.002). ALT and AST levels were not significantly different (AST (U/L): 103 ± 17 v.s. 111 ± 14, *p* = 0.355; ALT (U/L): 57 ± 7 v.s. 53 ± 24, *p* = 0.730). The results supported that rats fed with HFD for 8 weeks developed histology and biochemistry features of NAFLD.

**Figure 2:**
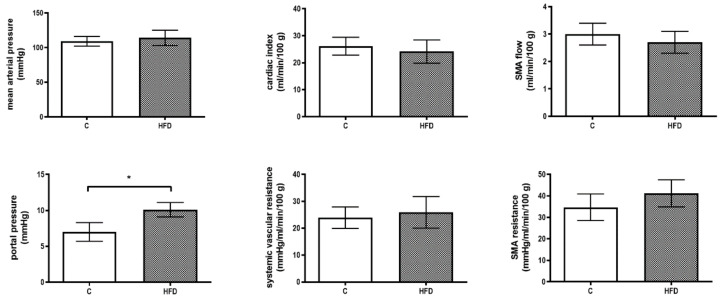
Effects of high fat diet (HFD) on hemodynamic parameters. Rats received regular chow diet (control, C) or HFD for 8 weeks. Portal pressure (PP) elevated significantly in HFD group. There was no significant difference in mean arterial pressure (MAP), cardiac index (CI), systemic vascular resistance (SVR), superior mesenteric artery (SMA) flow and resistance (* *p* < 0.05).

Figure 2 reveals the hemodynamic parameters. There was no significant difference in MAP, CI, SVR, SMA flow and SMA resistance between the two groups (MAP (mmHg): 109 ± 7 v.s. 114 ± 11, *p* = 0.364; CI (mL/min/100 g): 26.1 ± 3.3 v.s. 24.1 ± 4.3, *p* = 0.359; SVR (mmHg/mL/min/100 g): 23.9 ± 4.0 v.s. 25.9 ± 5.9, *p* = 0.470; SMA flow (mL/min/100 g): 3.0 ± 0.4 v.s. 2.7 ± 0.4, *p* = 0.104; SMA resistance (mmHg/mL/min/100 g): 34.7 ± 6.2 v.s. 41.2 ± 6.3, *p* = 0.075) except that PP increased significantly in HFD group (PP (mmHg): 7.0 ± 1.30 v.s. 10.1 ± 1.03, *p* < 0.001, *n* = 7, 7).

#### 3.1.2. Evaluation of HPA axis in NAFLD rats

**Figure 3:**
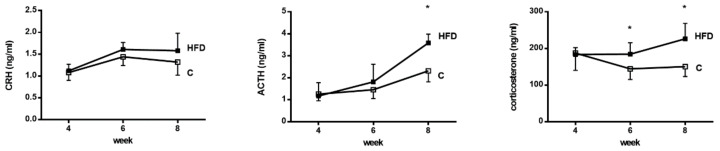
Influences of HFD on hypothalamus-pituitary-adrenal (HPA) axis in NAFLD rats. Plasma ACTH and corticosterone levels increased along with the time in HFD group. Nevertheless, corticotropin-releasing hormone (CRH) level was not significantly affected. (* *p* < 0.05).

The plasma CRH, ACTH and corticosterone levels were determined on the 4th, 6th and 8th week, respectively (Figure 3). CRH levels were not significantly different throughout the whole period of feeding (at the 8th week, CRH (ng/mL): 1.3 ± 0.3 v.s. 1.6 ± 0.4, *p* = 0.204, *n* = 7, 7). On the other hand, the plasma ACTH and corticosterone levels increased in HFD group (at the 8th week, ACTH (ng/mL): 2.3 ± 0.5 v.s. 3.6 ± 0.4, *p* < 0.001; corticosterone (ng/mL): 150 ± 27 v.s. 226 ± 42, *p* = 0.002).

### 3.2. Hemodynamic Response, Corticosterone Response and 11β-HSDs System Protein Expressions in Control and HFD Rats with LPS-Induced Sepsis

#### 3.2.1. The Hemodynamic Response to LPS Injection

**Figure 4:**
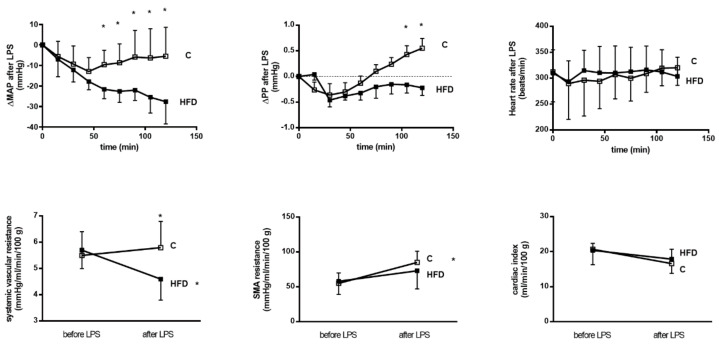
The hemodynamic response to lipopolysaccharide (LPS) injection. The changes of mean arterial pressure (ΔMAP), portal pressure (ΔPP) and HR (ΔHR) were monitored for 120 min after LPS injection. The MAP dropped at first in both groups. After 60 min, MAP was compensated in the control group but remained low in the HFD group. The ΔMAP was significantly higher in control group at 120 min. The PP changed in the same trend. The heart rate (HR) was not significantly different between the two groups. The systemic vascular resistance (SVR) and superior mesenteric artery (SMA) resistance were significantly lower in the HFD group after LPS injection (HFD group, before LPS v.s. after LPS; t-test). (* *p* < 0.05).

LPS was injected to simulate bacterial infection-related stress. MAP, PP and HR were monitored for 120 min after LPS administration (Figure 4). MAP dropped at the beginning of LPS administration in both groups. In control group, MAP elevated and was stabilized since 60 min after LPS injection. However, this was not observed in the HFD group. The drop of MAP was significantly more prominent in HFD group at 120 min ((mmHg): −5.5 ± 14.1 v.s. −27.6 ± 10.9, *p* = 0.002, *n* = 8, 9). At the same point, PP was elevated in control group but reduced in HFD group ((mmHg): 0.55 ± 0.55 v.s. −0.22 ± 0.44, *p* = 0.006). HR was not significantly different. The SVR, SMA resistance and CI were also measured before and at 120 min after LPS injection. SVR was not significantly different between the two groups before LPS injection. At 120 min, SVR was maintained in control group but decreased significantly in HFD group ((mmHg/mL/min/100 g): 5.8 ± 1.0 v.s. 4.6 ± 0.8, *p* = 0.020). The SMA resistance increased significantly in control group but not in HFD group (control group, before v.s. after LPS (mmHg/mL/min/100 g): 55 ± 15 v.s. 85 ± 16, *p* = 0.002). The CI was unaffected before and after LPS injection in both groups.

#### 3.2.2. The Corticosterone Response to LPS Injection

**Figure 5:**
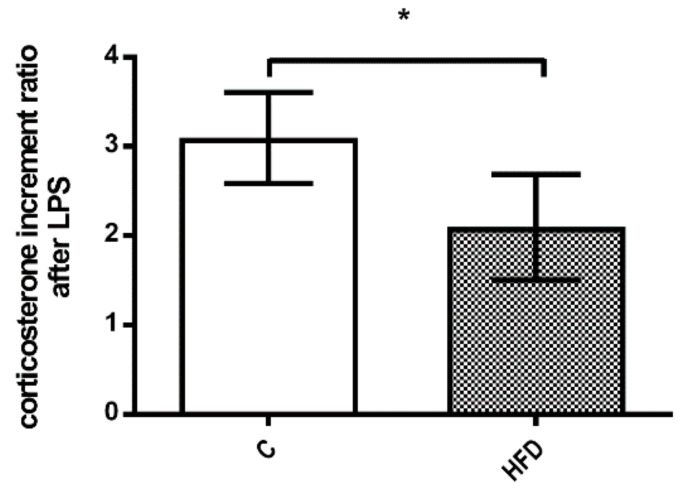
The corticosterone response to LPS injection. The HFD group had significantly lower corticosterone increment ratio after LPS injection. The results suggest inadequate corticosterone response to septic shock in NAFLD. (* *p* < 0.05).

The corticosterone increment ratio after LPS injection was significantly lower in HFD group (Figure 5, 3.1 ± 0.5 v.s. 2.1 ± 0.6, *p* = 0.021). The results suggest a poorer corticosterone response to septic shock in the HFD group.

### 3.3. Hemodynamic and Corticosterone Response to ACTH Stimulation in Control and HFD Rats with LPS-Induced Sepsis

#### 3.3.1. The Hemodynamic Response to ACTH Stimulation after Shock

**Figure 6:**
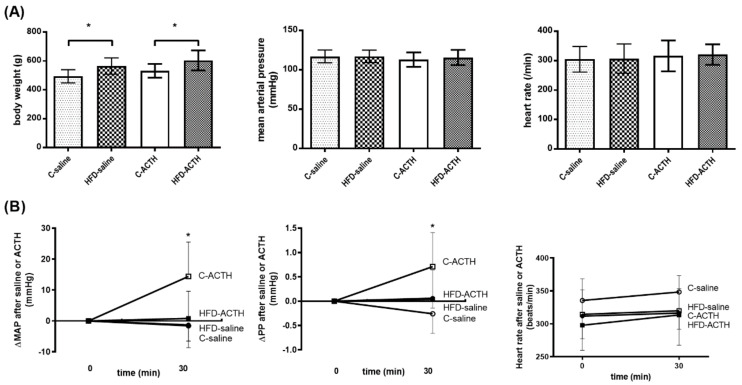
The hemodynamic response to adrenocorticotropic. hormone (ACTH) injection after LPS-induced septic shock. (**A**) The BW was significantly higher in HFD group. The MAP and HR was not significantly different among the four groups. (**B**) The ACTH or saline (served as vehicle) were given 120 min after LPS. The MAP increased significantly in C-ACTH group as compared to C-saline group at 30 min after ACTH injection (*t*-test). However, ACTH had no effect in HFD group. The PP showed consistent results (*t*-test). HR was not significantly different. (* *p* < 0.05).

ACTH or vehicle (saline) was injected, 120 min after LPS injection, to investigate the cardiovascular and corticosterone responses of control and HFD rats. The MAP, PP and HR were monitored for another 30 min after ACTH injection. Figure 6A reveals that the baseline hemodynamic parameters were not significantly different among the four groups. At 30 min after ACTH administration (Figure 6B), the elevation of MAP (ΔMAP) was not significantly different between saline- and ACTH-injected HFD groups. Besides, ΔMAP was significantly lower in HFD-ACTH group compared to C-ACTH group. (C-saline vs. HFD-saline vs. C-ACTH vs. HFD-ACTH: −1.6 ± 7.1 v.s. −1.3 ± 5.2 v.s. 14.4 ± 11.1 v.s. 0.8 ± 8.8, HFD-saline v.s. HFD-ACTH, *p* = 0.564; C-ACTH v.s. HFD-ACTH, *p* = 0.013). The ΔPP showed consistent results. There was no significant difference in HR throughout the whole experimental period.

#### 3.3.2. The Corticosterone Response to ACTH Stimulation after Shock

**Figure 7:**
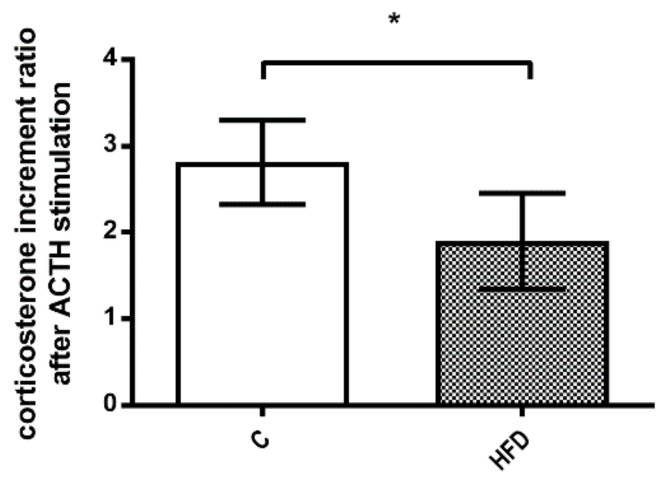
The corticosterone response to ACTH injection after LPS-induced septic shock. HFD group had a significant decrease in corticosterone increment ratio even after ACTH stimulation in septic shock status. (* *p* < 0.05).

Figure 7 depicts corticosterone response to ACTH stimulation after septic shock. The corticosterone increment ratio after ACTH stimulation was significantly lower in HFD group (2.8 ± 0.5 v.s. 1.9 ± 0.6, *p* = 0.025). This finding reinforced the impaired corticosterone response to septic shock and ACTH stimulation in NAFLD rats.

### 3.4. The Mechanistic Survey

#### 3.4.1. β-HSDs Protein Expressions in Adipose Tissue and Adrenal Gland without and with LPS-Induced Sepsis

**Figure 8:**
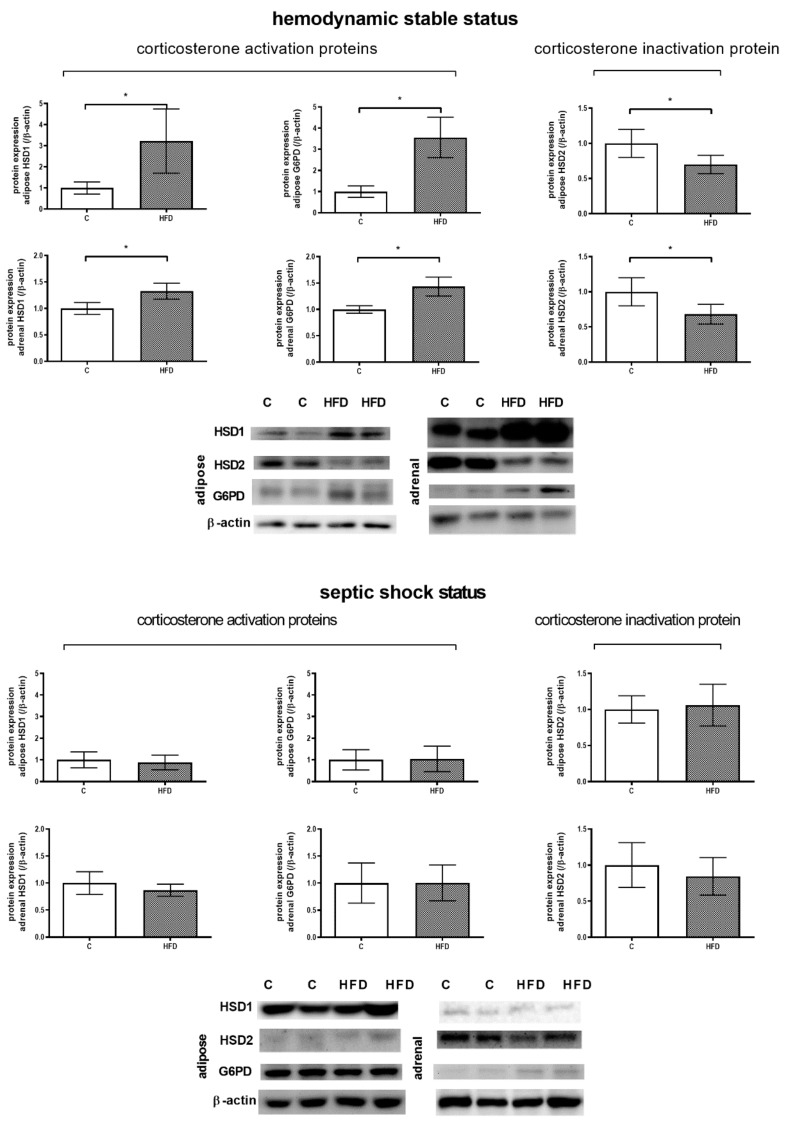
Adipose tissue and adrenal gland 11β-HSDs system-related proteins expressions. (**A**) 11β-HSD1, G6PD and 11β-HSD2 protein expressions in hemodynamic stable condition. In the adipose tissue and adrenal gland, the expressions of 11β-HSD1 and G6PD increased significantly whereas the expression of 11β-HSD2 reduced. (**B**) 11β-HSD1, G6PD and 11β-HSD2 protein expressions in septic shock status. The 11β-HSD system were not significantly different between the control and HFD groups. Taken together, the 11β-HSD system tends to produce more corticosterone in HFD rats in usual hemodynamic stable status but lost this trend in sepsis. This explains, at least partly, the failure of the NAFLD rats to generate enough corticosterone to combat shock. (* *p* < 0.05).

The balance between corticosterone (active form in rats) and its inactive form is regulated by 11β-HSD isoenzymes. While G6PD help 11β-HSD1 facilitate corticosterone production, 11β-HSD2 exerts the opposite function. Figure 8A discloses the protein expressions in different tissues in rats with relatively stable condition without sepsis. In adipose tissue and adrenal gland, 11β-HSD1 and G6PD expressions increased markedly and 11β-HSD2 expression decreased (adipose, 11β-HSD1: 1.00 ± 0.29 v.s. 3.22 ± 1.52, *p* = 0.008; 11β-HSD2: 1.00 ± 0.20 v.s. 0.70 ± 0.13, *p* = 0.007; G6PD: 1.00 ± 0.27 v.s. 3.56 ± 0.96, *p* < 0.001; adrenal gland, 11β-HSD1: 1.00 ± 0.11 v.s. 1.33 ± 0.15, *p* = 0.001; 11β-HSD2: 1.00 ± 0.20 v.s. 0.68 ± 0.14, *p* = 0.005; G6PD: 1.00 ± 0.07 v.s. 1.43 ± 0.18, *p* < 0.001). These results suggested that the up-regulations of 11β-HSD1, 11β-HSD2 and G6PD in NAFLD enhanced corticosterone production when hemodynamic condition was relatively stable. Figure 8B reveals the 11β-HSD system-related protein expressions in rats injected with LPS. Although in stable condition without sepsis, the 11β-HSDs system in the adipose tissue and adrenal gland acts toward corticosterone synthesis in HFD group, the trend changed after sepsis: The 11β-HSD1, G6PD, 11β-HSD2 protein expressions were not significantly different between the control and HFD groups. This explains, at least partly, the failure of the NAFLD rats to generate enough corticosterone to combat shock.

#### 3.4.2. The Vascular Vasoactive Substances Protein Expressions

COX1, COX2, iNOS, eNOS and phospho-eNOS protein expressions of aorta and SMA are presented in supplementary Figure S1A,B, showing that the aorta and SMA iNOS protein expressions were up-regulated in HFD group with LPS-induced sepsis (SMA iNOS: 1.00 ± 0.26 v.s. 1.45 ± 0.14, *p* = 0.001; aorta iNOS: 1.00 ± 0.13 v.s. 1.16 ± 0.17, *p* = 0.078, *n* = 7, 8).

## 4. Discussion

Animal models including genetic deficiencies, food, toxin or surgery have been developed to induce NAFLD or NASH. Genetic deficiency models are mostly applied to mice, such as ob/ob mice, db/db mice, SREBP-1c mice and so on. Regarding diet-modification methods, MCD diet (methionine and choline-deficiency), CD diet (choline-deficiency), intragastric overfeeding, high sucrose diet and high fat diet have been used. Each method has different limitations: MCD diet-fed rats do not develop obesity (they even get weight loss) and insulin resistance; CD diet induces liver steatosis and fibrosis without significant weight change; intragastric overfeeding needs specific training and equipment; high sucrose diet may induce mild liver inflammation without significant steatohepatitis and fibrosis; high fat diet may require longer feeding period to induce fatty liver diseases. Among them, high fat diet may be the most feasible one to simulate the lifestyle and intake pattern of NAFLD or NASH patients. A previous research from Japan revealed that 35% high fat diet (about 60% energy from fat) induced hepatic steatosis in four weeks [19].

The current study demonstrates that the weight of HFD rats was significantly higher than the control rats since the third week of feeding. In addition, HFD rats had higher blood glucose and total cholesterol levels at the 4th, 6th and 8th week and higher plasma insulin concentration and HOMA-IR level, the index of insulin resistance at the 8th week, supporting that HFD successfully induced features of metabolic syndrome. There were no significant differences of plasma aldosterone levels at the 4th, 6th and 8th week and also in MAP, HR and CO. The findings suggest that HFD feeding for 8 weeks does not significantly influence systemic hemodynamics.

The HFD rats had higher PP. Similarly, a previous study with rats fed with a cafeteria diet (CafD; 65% of fat, mostly saturated) for 1 month developed features of metabolic syndrome including overweight, arterial hypertension, hypertriglyceridemia, hyperglycemia, insulin resistance and liver steatosis without inflammation or fibrosis. Furthermore, liver perfusion demonstrated that CafD rats had an increased portal perfusion pressure and decreased intrahepatic vasodilation [20]. It is worth noting that in our current study, PP was significantly elevated in HFD-fed rats. In noncirrhotic condition, PP is determined mainly by two factors: splanchnic blood flow (indexed by SMA flow) and intrahepatic resistance. Since HFD did not significantly influence the SMA flow, in accordance with the previous study [20], the increased intrahepatic resistance may play a major role in HFD-induced portal hypertension.

The effects of NAFLD on baseline HPA axis activity is controversial [21]. In a systemic review included 20 original mouse studies, plasma corticosterone level was unaffected in 40% of studies. However, 30% of studies reported increased baseline corticosterone levels and 20% of studies showed decreased levels [22]. The inconsistent results may be due to different mouse strains, experiment design and/or content of diets. Interestingly, some human studies suggest that NAFLD may results in cortisol overproduction. Overweight T2DM individuals with NAFLD have significantly higher urine free cortisol level as compared to those without hepatic steatosis [4]. The same group also published that NAFLD patients have a subtle, chronic over-activity of the HPA axis leading to subclinical cortisol overproduction [5]. In this study, the unstimulated plasma corticosterone levels in usual status increased in NAFLD rats, which is consistent with human observation. Moreover, lower corticosterone increment ratio in septic shock in HFD rats were noted. The results indicated that HFD rats had higher baseline HPA axis activity but poorer corticosterone reactivity in response to septic shock.

There were higher protein expressions of 11β-HSD1 and G6PD and a lower protein expression of 11β-HSD2 in visceral adipose tissue and adrenal gland in HFD rats were noted. The major enzyme activity of 11β-HSD1 is exerted by oxo-reductase, which transfers 11-DHC to corticosterone by NADPH generated by G6PD or H6PDH [23,24]. Our results indicate that the up-regulated HPA axis activity in HFD rats may result from the enhanced 11β-HSD1 and G6PD expressions in visceral adipose tissue and adrenal gland. The 11β-HSD2 down-regulation also plays a role.

It has been reported that the incidence of sepsis is significantly higher in severely burned pediatric patients that has fatty infiltration of the liver [25]. Hepatic steatosis has also been noted in patients with sepsis but a causative relationship had not been established [26]. In addition, an animal experiment demonstrated that mice fed with HFD had a higher mortality rate when challenged with Staphylococcus aureus compared with those fed with a low fat diet [27]. Our current findings provide the evidence that HFD rats with liver steatosis exerted profound shock in response to LPS challenge and a worse corticosterone reactivity in response to ACTH stimulation. Furthermore, although in HFD rats under stable condition the adipose tissue and adrenal gland 11β-HSD system favors corticosterone synthesis, the condition alters after LPS administration: The 11β-HSD1, G6PD, 11β-HSD2 protein expressions were not significantly different between the control and HFD groups. This explains, at least partly, the failure of the HPA axis to generate enough corticosterone to ameliorate septic shock in rats with NAFLD.

The limitations of this study are as the followings: The 11β-HSD1 and G6PD protein expressions were upregulated in the adipose tissue and adrenal gland while 11β-HSD2 expression was down-regulated in the HFD group. However, the specific cell type involved in the changes are not clear. In addition, the underlying mechanism ascribed for septic shock-induced 11β-HSD system dysregulation deserves further clarification. Taken together, more investigations are required.

## 5. Conclusions

In conclusion, the current study reveals that HFD rats has a higher HPA axis activity, which is, at least partly, resulted from the up-regulation of 11β-HSD1 and G6PD and down-regulation of 11β-HSD2 in visceral adipose tissue and adrenal gland. HFD rats had profound shock in response to LPS challenge related to a poorer corticosterone reactivity and excess vascular iNOS production, which could be ascribed to the dysregulation of 11β-HSDs system in sepsis.

## Supplementary Materials

The following are available online at https://www.mdpi.com/1999-4923/12/5/403/s1, Figure S1: The vascular vasoactive substances protein expressions. (A, B) COX1, COX2, iNOS, eNOS and phospho-eNOS protein expressions of aorta and SMA. The aorta and SMA iNOS protein expressions were up-regulated in HFD group with LPS-induced sepsis. (* *p* < 0.05).
