# Supplementary material for: NAFLD Aggravates Septic Shock Due to Inadequate Adrenal Response and 11β-HSDs Dysregulation in Rats
Source: Pharmaceutics. 2020 Apr 28;12(5):403. doi: 10.3390/pharmaceutics12050403 (PMC7285211; doi:10.3390/pharmaceutics12050403)
Supplement: Supplementary file 1 [file pharmaceutics-12-00403-s001.pdf]

pharmaceutics

Supplementary Materials: NAFLD Aggravates Septic Shock Due to Inadequate Adrenal Response and 11β-Hsds Dysregulation in Rats

Hui-Chun Huang, Ming-Hung Tsai, Fa-Yauh Lee, Te-Yueh Lin, Ching-Chih Chang, Chiao-Lin Chuang, Shao-Jung Hsu, Ming-Chih Hou and Yi-Hsiang Huang

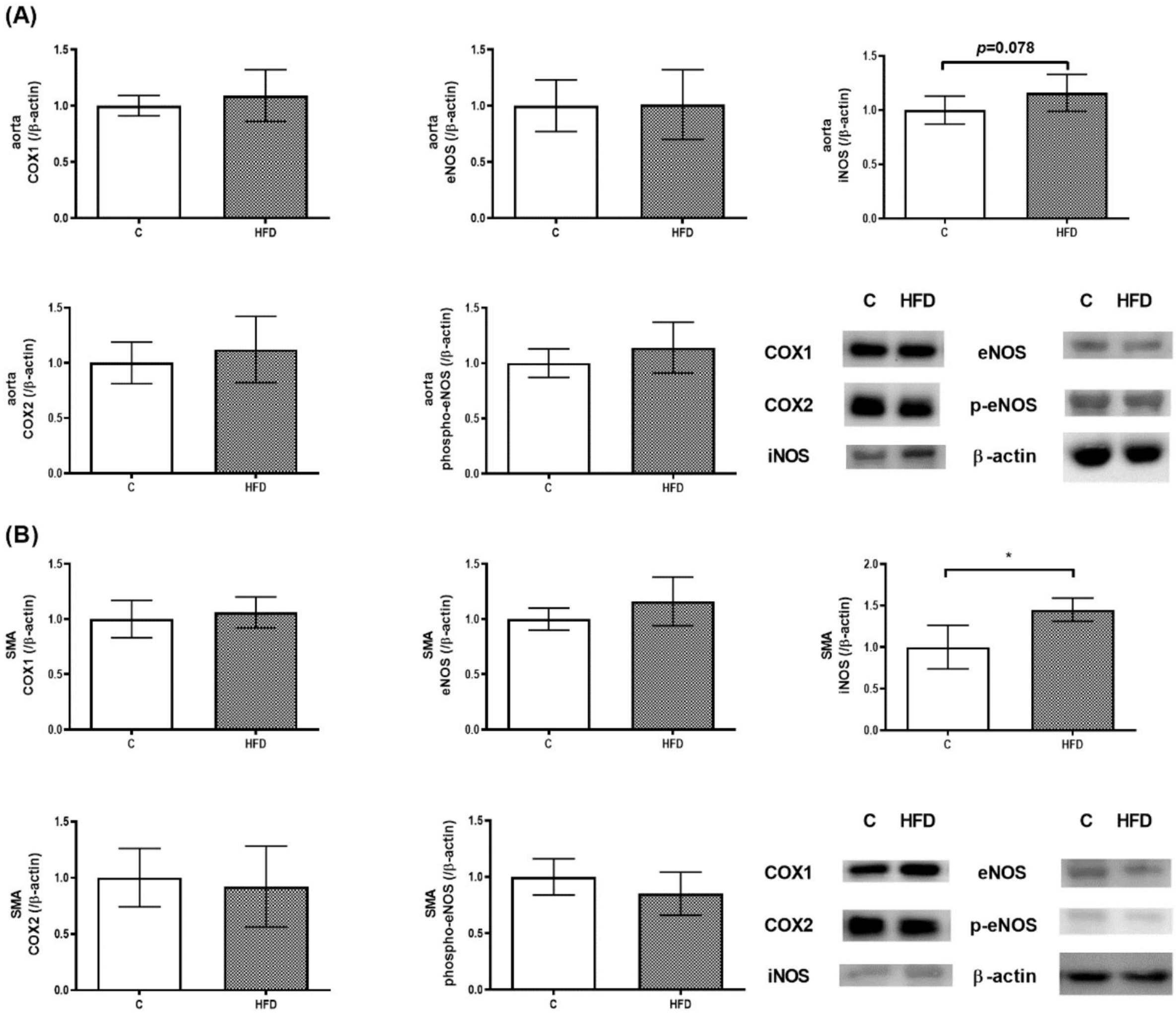

Figure S1. The vascular vasoactive substances protein expressions. (**A**, **B**) COX1, COX2, iNOS, eNOS and phospho-eNOS protein expressions of aorta and SMA. The aorta and SMA iNOS protein expressions were up-regulated in HFD group with LPS-induced sepsis. (* $p < 0.05$).
